# Biomarkers for Macrosomia Prediction in Pregnancies Affected by Diabetes

**DOI:** 10.3389/fendo.2018.00407

**Published:** 2018-07-31

**Authors:** Sofia Nahavandi, Jas-mine Seah, Alexis Shub, Christine Houlihan, Elif I. Ekinci

**Affiliations:** ^1^Department of Medicine, The University of Melbourne, Melbourne, VIC, Australia; ^2^Department of Endocrinology, Austin Health, Melbourne, VIC, Australia; ^3^Mercy Hospital for Women, Mercy Health, Melbourne, VIC, Australia

**Keywords:** diabetes, pregnancy, macrosomia, birthweight, biomarkers

## Abstract

Large birthweight, or macrosomia, is one of the commonest complications for pregnancies affected by diabetes. As macrosomia is associated with an increased risk of a number of adverse outcomes for both the mother and offspring, accurate antenatal prediction of fetal macrosomia could be beneficial in guiding appropriate models of care and interventions that may avoid or reduce these associated risks. However, current prediction strategies which include physical examination and ultrasound assessment, are imprecise. Biomarkers are proving useful in various specialties and may offer a new avenue for improved prediction of macrosomia. Prime biomarker candidates in pregnancies with diabetes include maternal glycaemic markers (glucose, 1,5-anhydroglucitol, glycosylated hemoglobin) and hormones proposed implicated in placental nutrient transfer (adiponectin and insulin-like growth factor-1). There is some support for an association of these biomarkers with birthweight and/or macrosomia, although current evidence in this emerging field is still limited. Thus, although biomarkers hold promise, further investigation is needed to elucidate the potential clinical utility of biomarkers for macrosomia prediction for pregnancies affected by diabetes.

## Introduction

With the increasing prevalence amongst women of childbearing age, diabetes mellitus is one of most common pre-existing medical conditions affecting pregnancy (1). Together, type 1 diabetes mellitus (T1DM) and type 2 diabetes mellitus (T2DM) affect around 1% of pregnancies (2). Gestational diabetes mellitus (GDM) is also on the rise, particularly since the changes in diagnostic criteria (3), with a prevalence of around 13% (4). This is of concern as maternal diabetes increases the risks associated with pregnancy (5, 6).

While there are higher rates of many adverse pregnancy outcomes, abnormal fetal growth and birthweight is particularly important due to the substantial frequency of occurrence (7, 8). Indeed, reports estimate macrosomia occurs in up to 60% of pregnancies affected by pre-existing diabetes (9). While macrosomia is common and occurs even in otherwise uncomplicated diabetic pregnancies (10), fetal growth restriction (FGR) and small-for-gestational-age (SGA) tend to occur in diabetic pregnancies complicated by other conditions, such as underlying maternal vascular disease (11, 12). For this reason, macrosomia in pregnancies affected by diabetes will form the focus of this review.

Macrosomia is defined in various ways. One method of defining macrosomia is according to the absolute birthweight being equal to or above a certain threshold, usually 4,000 g (13). Another definition is according to a birthweight percentile that accounts for the gestational age at birth, with macrosomia commonly defined as being above the 90th centile (also called large-for-gestational-age, LGA)(13, 14). The term macrosomia used here will encompass both of these definitions. Where possible, the abbreviated definition used by a referenced study will be mentioned, with the full definition provided in the supplementary table (Supplementary Table 1).

Given the potentially serious consequences for the mother and child, there is significant interest in predicting fetal macrosomia (13). Accurate identification holds potential for guiding appropriate management and interventions, with the aim of improving outcomes (15). However, currently available methods of macrosomia prediction demonstrate only modest predictive ability, which limits their use in tailoring obstetric decisions (13, 15).

The latest area of interest for potentially improving macrosomia prediction has been in the field of biomarkers. A biomarker in this context refers to a biological molecule that can be objectively assessed as an indicator of a physiological or pathological process or state, and therefore may have potential value for predicting certain outcomes (16, 17). A number of biomarkers have been investigated to determine whether they have an association with birthweight and macrosomia. Relevant biomarkers have been purposefully selected for further discussion.

The aim of this review is to examine the available literature for a relationship between selected biomarkers and birthweight and/or macrosomia. Pregnancies without diabetes in addition to pregnancies affected by diabetes will be discussed for comparison. The review will provide a brief overview of the determinants of fetal growth, the need for macrosomia prediction, and current prediction strategies. It will then focus on the selected biomarkers and provide evaluation of their birthweight/macrosomia prediction potential.

## Methods

Medline, Embase, and PubMed databases were searched in 2017. The following subject headings (and synonyms) were combined: pregnancy, diabetes mellitus, type 1 diabetes, type 2 diabetes, gestational diabetes, birthweight, fetal weight, macrosomia, large-for-gestational-age, biomarker, predictor. Specific searches also included the terms blood glucose, glycosylated hemoglobin, 1,5-anhydroglucitol, lipids, adiponectin, and insulin-like growth factor-1, as well as a search without diabetes terms. In addition, bibliographies of collected publications were manually searched.

Articles were selected if a biomarker from a maternal or fetal/neonatal biological sample was tested for an association with any measure of birthweight or macrosomia. This search identified a list of previously studied biomarkers (Table 1). Exclusion criteria included samples taken from pregnancies affected by FGR/SGA, multiple pregnancy, non-human studies, conference abstracts, and non-English articles.

**Table 1:** Biomarkers investigated for an association with birthweight or macrosomia (excluding FGR/SGA).

**Biomarker (Alphabetical order)**	**Source**	**Significant association with birthweight/macrosomia (Most adjusted result used. Maternal diabetes status of sample population provided; all were pregnant unless otherwise stated)**	**Non-significant association with birthweight/macrosomia (Most adjusted result used. Maternal diabetes status of sample population provided; all were pregnant unless otherwise stated)**
1,5-Anhydroglucitol	•Maternal blood	(18) T1DM, T2DM, GDM (19) T1DM, T2DM, GDM (20) T1DM	(21) GDM, No diabetes
25-Hydroxyvitamin D (25(OH)D)	•Maternal blood		(22) T2DM, GDM, No diabetes
32-33 Split proinsulin	•Umbilical cord blood	(23) T1DM, No diabetes	
Acid-Labile Subunit (ALS)	•Umbilical cord blood	(24) Diabetes status not stated	
Acylation Stimulating Protein (ASP)	•Umbilical cord blood	(25) No diabetes (pregnant), No diabetes (non-pregnant)	
Adiponectin	•Maternal blood	(26) GDM, No diabetes (27) GDM, No diabetes (pregnant), No diabetes (non-pregnant) (28) GDM, No diabetes (29) GDM, No diabetes (30) GIGT, No diabetes (31) No diabetes (32) No diabetes (33) No diabetes (31) No diabetes (34) No diabetes (34) No diabetes (35) No diabetes	(36) GDM, No diabetes (37) GDM, No diabetes (38) No diabetes (39) No diabetes
	•Umbilical cord blood	(36) GDM, No diabetes (40) GDM, No diabetes (32) No diabetes (39) No diabetes (41) No diabetes (42) No diabetes (43) No diabetes (44) No diabetes (45) No diabetes	(23) T1DM, No diabetes (46) T2DM, GDM, No diabetes (47) No diabetes (48) No diabetes (38) No diabetes
	•Amniotic fluid		(49) Diabetes status not stated
Albumin	•Amniotic fluid	(50) GDM, No diabetes	
Alpha-Feto Protein (AFP) ratio (maternal serum AFP / amniotic fluid AFP)	•Maternal blood •Amniotic fluid	(51) Diabetes status not stated	
Alpha Human Chorionic Gonadotropin (α-hCG)	•Maternal blood		(52) IDDM, No diabetes
Amino acids	•Umbilical cord blood		(53) IDDM, No diabetes
Anti-insulin antibodies	•Maternal blood •Cord blood		(54) IDDM, GDM, No diabetes
Apelin	•Maternal blood •Cord blood	(54) GDM, No diabetes	
Apolipoprotein A1 (ApoA1)	•Maternal blood		(30) GIGT, No diabetes (55) No diabetes
Apolipoprotein A5 (APOA5) S19W polymorphism	•Umbilical cord blood	(56) Diabetes status not-stated	
Apolipoprotein B (ApoB)	•Maternal blood		(30) GIGT, No diabetes (55) No diabetes
Aspartate aminotransferase	•Maternal blood	(57) No diabetes	
Beta Human Chorionic Gonadotrophin (β-hCG)	•Maternal blood	(58) Diabetes, No diabetes	(59) No diabetes
Beta-Hydroxybutyrate (β-OHB)	•Maternal blood	(60) Diabetes, No diabetes	
Bilirubin	•Maternal blood	(57) No diabetes	
Carcinoembryonic Antigen (CEA)	•Maternal blood •Umbilical cord blood		(61) Diabetes status not stated
Chemerin	•Umbilical cord blood	(62) GDM, No diabetes	
Coenzyme Q10 (CoQ10 or ubiquinone)	•Amniotic fluid	(63) GDM, No diabetes	
Copeptin	•Umbilical cord blood	(64) Diabetes, No diabetes	
Cortisol	•Maternal saliva	(65) No diabetes (66) Diabetes status not stated	
	•Amniotic fluid	(67) No diabetes	
C-peptide	•Umbilical cord blood	(53) IDDM, No diabetes (48) No diabetes (68) No diabetes	
	•Amniotic fluid		(69) Diabetes status not stated
C-Reactive Protein (CRP)	•Maternal blood	(30) GIGT, No diabetes (34) No diabetes	
Creatinine	•Maternal blood	(70) T1DM (57) No diabetes	
Cytokines: Interleukin (IL) IL-β, IL-6, IL-8	•Maternal blood		(71) No diabetes
Epidermal Growth Factor (EGF)	•Umbilical cord blood		(72) IDDM, GDM, No diabetes
	•Amniotic fluid		(73) Diabetes status not stated
E-selectin	•Maternal blood	(74) T1DM, T2DM	
Estriol	•Maternal blood	(38) No diabetes	
	•Umbilical cord blood		(38) No diabetes
Estradiol	•Maternal blood	(57) No diabetes	
Free thyroxine (FT4)	•Maternal blood	(75) No diabetes	
Fructosamine	•Maternal blood	(76) IDDM (77) Diabetes (type not specified) (54) IDDM, GDM, No diabetes (78) Pre-existing diabetes, GDM, No diabetes (79) GDM, No diabetes	(80) Pre-existing diabetes, GDM, No diabetes (81) T2DM, GDM (82) GDM, GIGT, No diabetes
	•Umbilical cord blood	(78) Pre-existing diabetes, GDM, No diabetes	
Fat mass- and obesity- associated (*FTO)* gene mRNA	•Placenta	(83) No diabetes	
Ghrelin	•Neonatal blood	(84) GDM, No diabetes (85) No diabetes	
Glucagon-like peptide 1 (GLP-1) - active	•Maternal blood	(86) No diabetes	
Glucose	•Maternal blood	(87) Pre-existing diabetes (88) IDDM (89) IDDM (90) T1DM (91) T1DM (92) T1DM (93) T1DM, No diabetes (94) GDM, GIGT, No diabetes (95) GDM, GIGT, No diabetes (96) GDM, No diabetes (97) GDM, No diabetes (98) GDM, No diabetes	(109) IDDM (110) GDM (111) GDM (25) No diabetes (pregnant), No diabetes (non-pregnant)
		(99) GDM, No diabetes (100) GDM, No diabetes (101) GDM (30) GIGT, No diabetes (102) No diabetes (103) No diabetes (104) No diabetes (105) No diabetes (106) No diabetes (107) No diabetes (108) No diabetes	
	•Umbilical cord blood	(48) No diabetes	
	•Amniotic fluid		(112) No diabetes
	•Maternal urine	(113) No diabetes	
Glycated albumin	•Maternal blood	(114) GDM, No diabetes	
Glycine/valine ratio	•Amniotic fluid		
Glycosylated hemoglobin (HbA1c)	•Maternal blood	(115) Pre-existing diabetes, GDM (116) Pre-existing diabetes, No diabetes (109) Pre-existing diabetes (117) IDDM, GDM, ‘Probably normal’, Normal (118) IDDM, No diabetes (119) IDDM (120) IDDM, GDM, No diabetes (121) T1DM, T2DM (122) T1DM, T2DM (81) T2DM, GDM (123) T1DM (124) T1DM (20) T1DM (125) T1DM (126) T1DM (127) T1DM (91) T1DM (128) T1DM (90) T1DM (92) T1DM (129) T1DM, No diabetes (130) No diabetes (131) No diabetes (103) No diabetes (132) No diabetes	(87) Pre-existing diabetes (88) IDDM (18) T1DM, T2DM, GDM (19) T1DM, T2DM, GDM (133) T1DM (95) GDM, IGT, No diabetes (134) GDM, IGT, No diabetes (21) GDM, No diabetes (111) GDM (135) GDM (136) GIGT, No diabetes (137) No diabetes (pregnant), No diabetes (non-pregnant) (138) No diabetes (139) No diabetes
	•Umbilical cord blood		(68) No diabetes
Glycosylated proteins	•Maternal blood	(140) T1DM, No diabetes (103) No diabetes	(118) IDDM, No diabetes
	•Umbilical cord blood	(140) T1DM, No diabetes	
Growth factor receptor-bound protein 10 *(GRB10)* gene single nucleotide polymorphism rs12540874 A>G	•Placenta	(141) No diabetes	
Growth Hormone Binding Protein	•Maternal blood		(142) IDDM, NIDDM, No diabetes
HDL-Cholesterol (HDL-C)	•Maternal blood	(143) T1DM, T2DM, No diabetes (111) GDM (144) GDM
		(145) GDM, No diabetes (100) GDM, No diabetes (146) No diabetes (55) No diabetes (146) No diabetes (147) No diabetes	(21) GDM, No diabetes (30) GIGT, No diabetes (57) No diabetes
	•Umbilical cord blood	(43) No diabetes	(148) T1DM, No diabetes (68) No diabetes
Hepatocyte Growth Factor (HGF)	•Amniotic fluid		(149) No diabetes
Homocysteine	•Maternal blood •Umbilical cord blood	(150) Diabetes status not stated	
Insulin	•Maternal blood		(30) GIGT, No diabetes
	•Umbilical cord blood	(151) IDDM, No diabetes (23) T1DM, No diabetes (62) GDM, No diabetes (64) Diabetes, No diabetes (43) No diabetes (152) No diabetes (48) No diabetes (68) No diabetes	
	•Amniotic fluid	(153) IDDM, GDM, No diabetes (154) T1DM, T2DM, GDM, GIGT, No diabetes (155) T1DM	(69) Diabetes status not stated
Insulin-like Growth Factor-1 (IGF-1)	•Maternal blood	(156) IDDM (142) IDDM, NIDDM, No diabetes (157) T1DM, T2DM, GDM, No diabetes (158) T1DM (159) GDM, No diabetes (160) Diabetes status not stated (161) No diabetes (162) No diabetes	(163) IDDM, No diabetes (164) T1DM, No diabetes (126) T1DM (165) GDM, No diabetes (166) Diabetes status not stated (38) No diabetes (167) No diabetes (168) No diabetes
	•Umbilical cord blood	(169) Pre-existing diabetes, GDM, No diabetes (170) IDDM, NIDDM, No diabetes (171) T1DM, T2DM, GDM (172) T1DM, GDM, No diabetes (173) T1DM, No diabetes (174) GDM, No diabetes (24) Diabetes status not stated (175) Diabetes status not stated (166) Diabetes status not stated (161) No diabetes (176) No diabetes	(177) T1DM, No diabetes (164) T1DM, No diabetes (38) No diabetes (178) No diabetes (168) No diabetes
Insulin-like Growth Factor-2 (IGF-2)	•Maternal blood	(142) IDDM, NIDDM, No diabetes (156) IDDM (158) T1DM (159) GDM, No diabetes	(159) GDM, No diabetes (168) No diabetes (176) No diabetes
	•Umbilical cord blood		(38) No diabetes (169) Pre-existing diabetes, GDM, No diabetes (168) No diabetes
Insulin-like Growth Factor Binding Protein-1 (IGFBP-1)	•Maternal blood	(142) IDDM, NIDDM, No diabetes	
	•Umbilical cord blood	(179) T1DM, T2DM, GDM (178) No diabetes	
Insulin-like Growth Factor Binding Protein-2 (IGFBP-2)	•Maternal blood		(142) IDDM, NIDDM, No diabetes
Insulin-like Growth Factor Binding Protein-3 (IGFBP-3)	•Maternal blood		(38) No diabetes (142) IDDM, NIDDM, No diabetes
	•Umbilical cord blood	(24) Diabetes status not stated	(38) No diabetes
Insulin-like Growth Factor-1 Receptor (*IGF1R)* mRNA	•Placenta	(180) GDM, No diabetes	
Interlukin-6 (IL-6)	•Umbilical cord blood		(48) No diabetes
Irisin	•Umbilical cord blood	(181) GDM, No diabetes	
LDL-Cholesterol (LDL-C)	•Maternal blood		(143) T1DM, T2DM, No diabetes (21) GDM, No diabetes (111) GDM (30) GIGT, No diabetes (55) No diabetes (57) No diabetes (146) No diabetes
	•Umbilical cord blood	(148) T1DM, No diabetes	(43) No diabetes (68) No diabetes
Leptin	•Maternal blood	(182) T1DM, No diabetes (30) GIGT, No diabetes (183) GDM, No diabetes (184) No diabetes	
	•Umbilical cord blood	(182) T1DM, No diabetes (184) No diabetes (43) No diabetes (185) No diabetes (41) No diabetes (47) No diabetes (178) No diabetes	(48) No diabetes
Lipoxin A_4_ (LXA_4_)	•Maternal blood	(186) GDM, No diabetes	
Metabolites: taurine, creatinine, betaine, glycine, citrate, myo-inositol	•Neonatal urine	(187) Diabetes status not stated	
MicroRNA-21 (miR-21)	•Placenta	(188) No diabetes	
MicroRNAs (miR): miR-141-3p, miR-200c-3p	•Maternal blood	(189) Diabetes status not stated	
MicroRNA-376a (miR-376a)	•Maternal blood	(190) No diabetes	
Mitochondrial DNA (mtDNA)	•Maternal blood		(191) GDM, No diabetes
Nesfatin-1	•Maternal blood		(54) GDM, No diabetes
	•Cord blood		(54) GDM, No diabetes
Obestatin	•Umbilical cord blood		(62) GDM, No diabetes
Pigment Epithelium-Derived Factor (PEDF)	•Umbilical cord blood	(46) T2DM, GDM, No diabetes	
Platelets	•Umbilical cord blood	(129) T1DM, No diabetes	
Placental Growth Factor (PlGF)	•Maternal blood	(192) Diabetes, No diabetes (193) Diabetes, No diabetes	
	•Amniotic fluid		(49) Diabetes status not stated
Placental Growth Hormone (PGH)	•Maternal blood	(142) Pre-existing IDDM, NIDDM, No diabetes (164) T1DM, No diabetes (158) T1DM (126) T1DM	(194) No diabetes (194) No diabetes
	•Umbilical cord blood		(164) T1DM, No diabetes
Placental imprinted genes: BLCAP, DLK1, H19, IGF2, MEG3, MEST, NNAT, NDN, PLAGL1	•Placenta	(195) Diabetes status not-stated	
Placental Lactogen	•Maternal blood	(57) No diabetes	
Placental Protein 13 (PP13)	•Maternal blood	(196) Diabetes status not stated	
Plasminogen Activator Inhibitor-type 1 (PAI-1)	•Maternal blood		(34) No diabetes
Plasminogen Activator Inhibitor-2 (PAI-2)	•Maternal blood	(192) Diabetes not excluded	
Pregnancy-Associated Plasma Protein-A (PAPP-A)	•Maternal blood	(58) Diabetes, No diabetes (197) Diabetes, No diabetes (198) No diabetes (199) Diabetes status not stated	(59) No diabetes
Progesterone	•Maternal blood	(38) No diabetes	
	•Umbilical cord blood	(38) No diabetes	
Prolactin	•Maternal blood		(38) No diabetes
Regulated on Activation, Normal T cell Express and Secreted upon uptake (RANTES)	•Umbilical cord blood	(200) T2DM, GDM, No diabetes	
Retinol-Binding Protein 4 (RBP4)	•Umbilical cord blood	(201) GDM, No diabetes	
Resistin	•Maternal blood		(202) GDM, No diabetes (34) No diabetes
	•Umbilical cord blood		(202) GDM, No diabetes
RNA: *PHLDB2, CLDN1, C15orf29, LPHN3, LEP, GCH1*,	•Placenta	(203) Diabetes status not stated	
Sex Hormone Binding Globulin (SHBG)	•Maternal blood		(38) No diabetes
	•Umbilical cord blood	(174) GDM, No diabetes	(38) No diabetes
Soluble Fms-like tyrosine kinase-1 (sFlt-1)	•Maternal blood	(192) Diabetes, No diabetes	
Soluble Fms-like tyrosine kinase-1 /Placental Growth Factor ratio	•Maternal blood	(193) No diabetes	
Soluble Leptin Receptor (sOB-R)	•Umbilical cord blood	(47) No diabetes	
Soluble TNF-α receptor-2 (TNFR2)	•Maternal blood •Umbilical cord blood	(35) No diabetes	
Squalene	•Maternal blood	(204) GDM, No diabetes	
Stromal Cell-derived Factor-1a (SDF-1a)	•Amniotic fluid	(205) No diabetes	
Testosterone	•Maternal blood		(38) No diabetes
	•Umbilical cord blood		(38) No diabetes
Total cholesterol	•Maternal blood		(111) GDM (21) GDM, No diabetes (30) GIGT, No diabetes
			(146) No diabetes (55) No diabetes
	•Umbilical cord blood	(206) GDM, No diabetes	(43) No diabetes (68) No diabetes
Total lipids	•Maternal blood		(111) GDM
Triglycerides	•Maternal blood	(143) T1DM, T2DM, No diabetes (94) GDM, GIGT, No diabetes (207) GDM, GIGT, No diabetes (208) GDM, No diabetes (209) GDM (144) GDM (210) GDM, No diabetes (146) No diabetes (211) No diabetes (212) No diabetes (213) No diabetes (146) No diabetes (214) No diabetes (25) No diabetes (pregnant), No diabetes (non-pregnant)	(148) T1DM, No diabetes (21) GDM, No diabetes (111) GDM (100) GDM, No diabetes (30) GIGT, No diabetes (215) No diabetes
	•Umbilical cord blood	(211) No diabetes (215) No diabetes	(43) No diabetes (55) No diabetes
Tumor Necrosis Factor-α (TNF-α)	•Maternal blood		(71) No diabetes
Uric acid	•Maternal blood		(55) No diabetes
Vascular Cell Adhesion Molecule-1 (sVCAM-1)	•Maternal blood	(74) T1DM, T2DM	
Vascular Endothelial Growth Factor (VEGF)	•Maternal blood	(193) No diabetes (216) Diabetes status not stated	
Very Low Density Lipoprotein (VLDL)	•Maternal blood	(57) No diabetes	
Visfatin	•Maternal blood	(48) No diabetes	
	•Umbilical cord blood		(48) No diabetes
Vitamin C	•Maternal blood •Umbilical cord blood	(217) No diabetes	

*T1DM, Type 1 diabetes mellitus; T2DM, Type 2 diabetes mellitus; GDM, Gestational diabetes mellitus; IDDM, Insulin-dependent diabetes mellitus; NIIDM, Non-insulin dependent diabetes mellitus; GIGT, Gestational impaired glucose tolerance*.

## Discussion

### Determinants of fetal growth

Normal fetal growth relies on the complex interplay of multiple factors, including genetic and environmental influences arising from the parents, fetus, and placenta (218). A key determinant of abnormal growth is altered substrate supply to the fetus (219). In normal pregnancy, maternal insulin resistance increases across gestation, becoming most pronounced in the third trimester when the majority of fetal growth takes place (220). This adaptive change promotes diversion of glucose across the placenta down its concentration gradient to the fetus (221). However, in pregnancies affected by diabetes, such transfer is exaggerated due to maternal hyperglycaemia (221). This excess glucose supply is believed to be central to diabetes-related fetal overgrowth. Indeed, the hyperglycaemia-hyperinsulinaemia hypothesis (also known as the Pedersen hypothesis), has been the prevailing explanation for macrosomia in diabetic pregnancies (220). It proposes that maternal hyperglycaemia leads to fetal hyperglycaemia, which stimulates maturation and hypertrophy of the fetal pancreas (222). This results in hypersecretion of insulin, and as insulin is a dominant fetal growth hormone, acceleration of fetal growth occurs (219). The modified Pedersen hypothesis also includes maternal amino acids and lipids in addition to glucose, contending that these insulin-responsive maternal fuels lead to an increase in “mixed nutrients” supplied to the fetus, which in turn elevates fetal insulin and drives excessive growth (220).

### Rationale for macrosomia prediction

Antenatal prediction of fetal macrosomia prediction is desirable for many reasons. Firstly, macrosomia is a common obstetric complication, affecting a significant number of pregnancies. According to recent Australian figures, the rate of macrosomia (birthweight ≥ 4,000 g) amongst pregnancies with pre-existing type 1 and type 2 diabetes was 25 and 18% for male and female offspring, respectively (2). However, as previously mentioned, other populations have reported rates up to 60% (for pre-existing diabetes), which is approximately six times the rate for women without diabetes (9).

**Figure 1:**
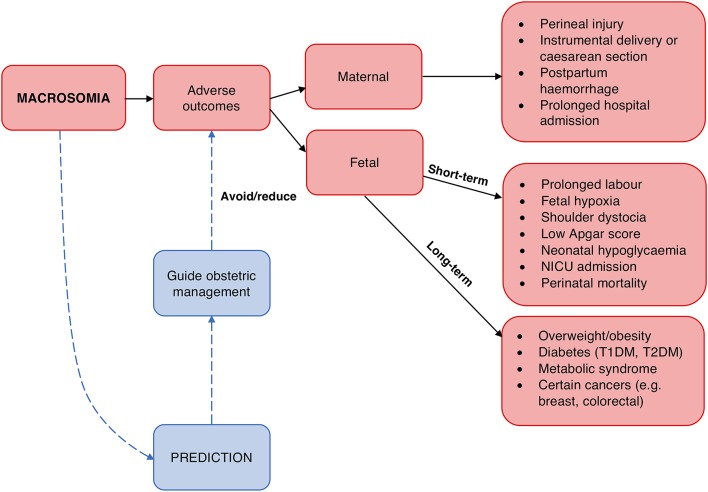
Rationale for macrosomia prediction. Macrosomia is associated with a number of adverse outcomes for both the mother and fetus (223–231). Prediction of macrosomia may reduce or avoid these via guiding appropriate obstetric management.

Secondly, macrosomia carries risks for the mother and fetus (Figure 1). Prominent risks include obstructed labor, cesarean section, instrumental delivery, perineal trauma, and birth injuries such as shoulder dystocia, an obstetric emergency involving difficulty delivering the fetal shoulders (14, 223, 224). Moreover, the risk of shoulder dystocia is greater in pregnancies affected by diabetes for any given birthweight, perhaps due to the altered fetal body proportions (14, 232). Longer-term risks include obesity and diabetes in the offspring (225, 226), possibly reflecting fetal programming as proposed by the “developmental origins of adult disease” or Barker hypothesis (233, 234).

By identifying pregnancies at increased perinatal risk, macrosomia prediction allows for tailoring obstetric care. Appropriate management and interventions could be employed to avoid or reduce the associated risks. Induction of labor and elective cesarean section are possible options; although, definitive evidence for improved outcomes and cost-effectiveness from these strategies is lacking at present (15, 235). Thus, further development and evaluation of appropriate management options is needed, and improved prediction strategies could be instrumental in this.

### Available methods for macrosomia prediction

Different methods for predicting macrosomia are currently available, as outlined in Table 2. Risk-factor based prediction aims to assess the likelihood of macrosomia based on identified unmodifiable and modifiable risk factors (13). Diabetes is the strongest risk factor for macrosomia (13, 235), and even maternal hyperglycaemia below diagnostic thresholds for diabetes increases the risk (102). Maternal body mass index (BMI) and gestational weight gain (GWG) are also well-established risk factors (252, 253), with pre-pregnancy obesity increasing the odds of macrosomia by threefold (254). Furthermore, clinical methods of fetal size estimation include physical examination techniques, with symphysis fundal height measurement and abdominal palpation being the primary manoeuvres (255). Maternal estimation of fetal weight by parous women has also been described (247). Finally, ultrasound estimation of fetal weight is routinely used, which employs formulae incorporating fetal biometric parameters (248).

**Table 2:** Evaluation of available methods for macrosomia prediction.

**Method**	**Description**	**Performance**	**Advantages**	**Disadvantages**
Risk-factor assessment	Assesses the likelihood of macrosomia based on factors known to increase macrosomia risk. Unmodifiable risk factors include maternal age, parity, parental height, ethnicity, fetal sex (male), and previous macrosomic delivery (224, 236–238). Modifiable risk factors include pre-pregnancy weight, GWG, gestational age, impaired glucose tolerance/diabetes (224, 236, 238, 239).	Accuracy varies with the risk factors assessed and population studied. One prediction equation demonstrated 57% sensitivity, 90% specificity, PPV 47%, NPV 93% (cut off value 3,750 g); although this excluded women with complications including diabetes (240).	Risk factors can be readily assessed with history and examination No cost Non-invasive	A validated, accessible, user-friendly predictive tool using risk factors is lacking A notable proportion of macrosomia occurs in pregnancies that have no or low identifiable risk
Symphysis fundal height (SFH) measurement	The SFH is a measurement of the maternal abdomen from the superior margin of the symphysis pubis to the highest point of the uterine fundus using a tape measure (241). Measurements greater than the normal range for gestational age as per fundal height curves may indicate a large fetus (241).	Estimates of the predictive performance for macrosomia vary widely, with reported sensitivity ranging from 16–98% and specificity of 88-95% (242–244).	Available at the bedside No cost Non-invasive	Accuracy problems relating to the measurement technique, inter-observer variability, gestational age dating or use of different fundal height curves. Maternal diabetes and obesity may also affect accuracy (245)
Abdominal palpation	Abdominal palpation using Leopold manoeuvres estimate fetal size by tactile assessment of fetal parts (246).	When performed by experienced clinicians, abdominal palpation can predict 70% of birthweights to within 10% of the actual value (246).	Available at the bedside No cost Non-invasive	Accuracy influenced by the subjective nature of the assessment and operator-dependence
Maternal estimation	A parous women is asked to estimate the birthweight of her child prior to delivery (247).	A study in post-term pregnancies demonstrated prediction of macrosomia with 56% sensitivity, 94% specificity, PPV 77%, NPV 86%(247).	Available at the bedside No cost Non-invasive Involves the mother	Limited to women with a previous pregnancy
Ultrasound assessment	Ultrasound assessment of fetal size involves determining the gestational age of the pregnancy, measurement of fetal biometry (e.g., abdominal circumference), use of various formulae to estimate fetal weight, and comparing fetal size with population standard charts for gestational age to obtain the corresponding percentile (248).	Masurement error of ultrasound fetal weight estimation has been reported as ± 15–20% (241, 249). Poorer accuracy at the extremes of fetal weight (250). Mean detection rate of macrosomia is 29% in the general obstetric population (250). Margin of error in pregnancies with diabetes is ±20–25% (251).	Wide availability Rapidly produces results Perceived objectivity	Requires trained operators Resource requirements & costs Inconvenience of extra appointments

*PPV, positive predictive value; NPV, negative predictive value*.

In comparing the performance of the available prediction methods, the broad conclusion is that no single method demonstrates clear superiority over the others (246, 256). Importantly, these current methods all have their limitations—a major limitation is their imprecision, displaying a sensitivity and specificity for macrosomia detection of around 55 and 90%, respectively (15, 246). The false positive and false negative rates are of concern, as inaccurate results can carry serious consequences including unnecessary intervention (14, 256, 257). Thus, these methods have limited clinical utility and caution is needed if used to guide management (15). From this it is evident there exists a need to improve macrosomia prediction beyond current capabilities, particularly in pregnancies affected by diabetes.

### Biomarkers for macrosomia prediction

Biomarkers may hold potential for enhancing macrosomia prediction. Biomarkers represent a biological source of information, revealing unique insight into the in-utero environment that may be leading to accelerated fetal growth. Hence by reflecting the possible proximal determinants of excessive growth, biomarkers could provide predictive capacity for macrosomia.

**Figure 2:**
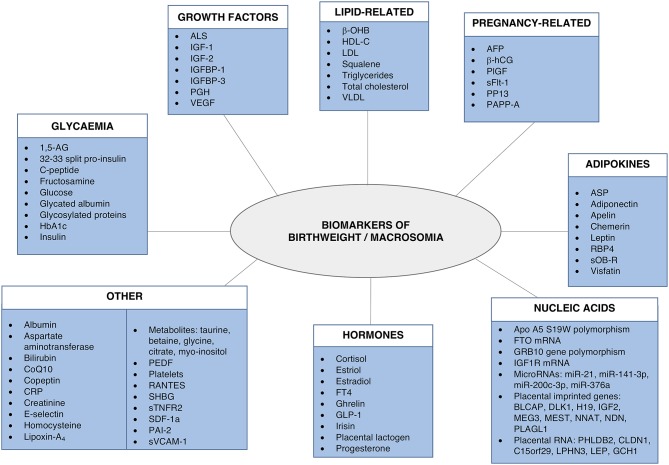
Biomarkers associated with birthweight and/or macrosomia. Biomarkers that have previously demonstrated a significant association with birthweight and/or macrosomia. Abbreviations provided in Table 1.

**Figure 3:**
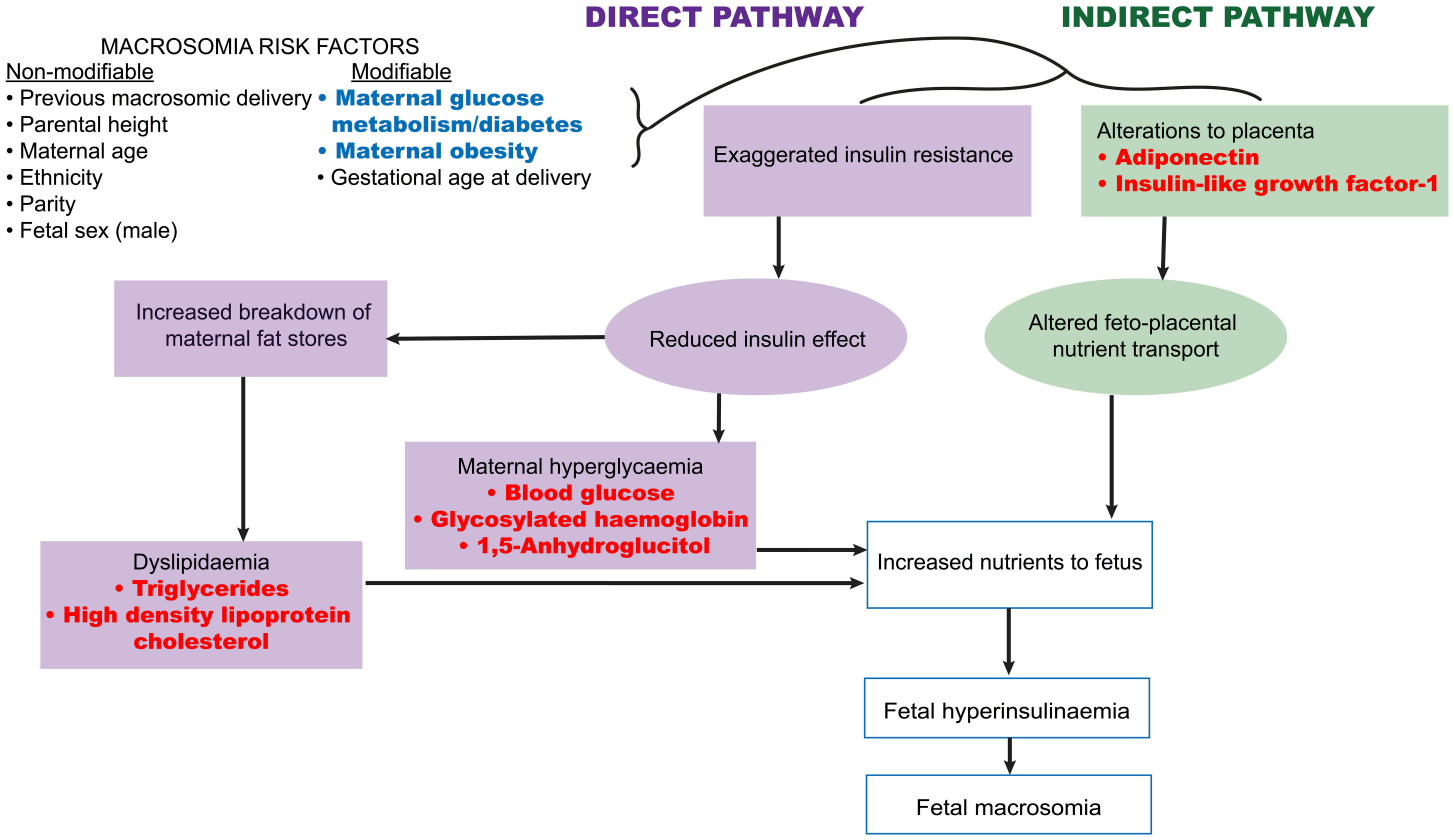
Proposed link between macrosomia risk factors and the selected biomarkers. Maternal diabetes and obesity have proposed links to fetal macrosomia via direct and indirect effects on fetal growth (249, 258). Biomarkers (red) possibly related to these pathways may therefore capture information that has predictive capacity for macrosomia.

A number of fetal and maternal biomarkers have been previously assessed for an association with birthweight or macrosomia in pregnancies with and without diabetes (Table 1;Figure 2). An approach to selecting biomarkers for further evaluation was informed by the known risk factors for macrosomia. The risk factors for which a detectable biological correlate (biomarker) may be present and therefore may reflect “proximal macrosomia determinants” are maternal glucose metabolism/diabetes and maternal weight (pre-pregnancy obesity and GWG). Although the underlying mechanisms by which these risk factors mediate their influence on fetal growth have not yet been definitively determined, a theory linking these two with fetal macrosomia considers “direct” and “indirect” pathways (249, 258) (Figure 3).

The direct pathway relates to insulin effectiveness and action (221, 249). Maternal diabetes and/or obesity affects this pathway via exaggerating the physiological insulin resistance that develops during pregnancy, which in-turn contributes to maternal hyperglycaemia and dyslipidaemia (249). This then leads to increased nutrient delivery to the fetus, subsequently resulting in fetal hyperinsulinaemia and macrosomia as per the modified Pedersen hypothesis (249, 258). Thus, biomarkers of maternal glycaemic control that may provide indication of the glycaemia-related risk of the direct pathway include blood glucose, glycosylated hemoglobin (HbA1c), and 1,5-anhydroglucitol (1,5-AG). While maternal triglycerides and cholesterol may be markers of the dyslipidaemia-related effects on growth. On the other hand, the indirect pathway centers on placental function (249). Placental changes that have been linked to maternal diabetes and obesity, such as alterations in structure, utero-placental blood flow, and placental transporters, may lead to altered feto-placental nutrient transport (249, 259). This likewise increases nutrient delivery to the fetus and stimulates fetal hyperinsulinaeamia. As adiponectin and insulin-like growth factor-1 (IGF-1) are proposed to be involved in these placental alterations, they therefore represent potential biomarkers of this indirect pathway (249). Hence these biomarkers are prime candidates to be assessed for associations with fetal weight. Maternal rather than fetal (cord blood) biomarkers will be the focus due to being the source relevant for antenatal predictive testing. Pre-existing diabetes, GDM, and pregnancies without diabetes will be examined in turn (results summarized in Table 3).

**Table 3:** Summary of evidence in support of an association between the selected biomarkers and birthweight/macrosomia.

**Biomarker (Maternal source unless otherwise stated)**	**T1DM**	**T2DM**	**GDM**	**No diabetes**
Blood glucose	Strongest evidence for second & third trimester measurements. Postprandial over fasting measurements.	Strongest evidence for second & third trimester measurements. Postprandial over fasting measurements.	Support for glucose parameters in the oral glucose tolerance test.	Support for glucose parameters in the oral glucose tolerance test.
Glycosylated hemoglobin	Strongest evidence for third trimester measurements.	Strongest evidence for third trimester measurements.	Limited supportive evidence.	Limited supportive evidence.
1,5-Anhydroglucitol	Significant association in all available studies.	Significant association in all available studies.	Mixed results.	No supportive evidence.
Lipids	Triglycerides and HDL-C with most support.	Triglycerides and HDL-C with most support.	Triglycerides and HDL-C with most support.	Triglycerides and HDL-C with most support.
Adiponectin	Lack of maternal adiponectin studies. Fetal adiponectin not significant (limited studies).	Lack of maternal adiponectin studies. Fetal adiponectin not significant (limited studies).	Some support for maternal and fetal adiponectin.	Some support for maternal and fetal adiponectin.
Insulin-like growth factor-1	Mixed results for maternal IGF-1. Stronger support for fetal IGF-1.	Mixed results for maternal IGF-1. Stronger support for fetal IGF-1.	Some support for maternal and fetal IGF-1.	Some support for maternal and fetal IGF-1.

#### Blood glucose

In women with pre-existing diabetes, various parameters of blood glucose have been assessed for associations with birthweight. The Diabetes in Early Pregnancy (DIEP) study focused on elucidating the contribution of fasting verses postprandial glucose to infant birthweight, comparing women with T1DM and controls across pregnancy (93). The findings indicated that postprandial glucose was more important for macrosomia (birthweight ≥ 90th percentile) risk, with the third trimester postprandial glucose levels the strongest predictor. Other studies also support the importance of postprandial blood glucose; with postprandial glucose levels in the third trimester predicting macrosomia (birthweight >90th percentile) (87), and mean postprandial glucose in the second trimester associated with birthweight (91). However, these studies have often analyzed the fasting and postprandial glucose measurements as the average across a whole trimester, hindering more specific timing effects of glycaemia on macrosomia risk to be determined. Addressing this by calculating the mean fasting and post-prandial glucose measurements over 3 week blocks in women with pre-existing diabetes, Persson et al. found the mean fasting glucose levels between 27 and 29 weeks' gestation were independently associated with macrosomia (birthweight >2 standard deviations), whereas postprandial levels were not (88). This fasting glucose measurement and pre-pregnancy weight together accounted for 12% of the variance in birthweight. In addition to the different time periods over which averages were calculated, variations in the measurement methods including the use of patient self-monitoring of blood glucose (SMBG) via a glucometer compared to laboratory testing, could have contributed to the conflicting findings. Nonetheless, they substantiate the contribution of second and third trimester maternal glycaemia to fetal growth in pregnancies complicated by pre-existing diabetes (89, 260, 261).

Furthermore, as postprandial hyperglycaemia involves transitory glycaemic excursions, this supports the notion that glucose fluctuations in addition to chronic hyperglycaemia, are important in influencing excessive fetal growth (92, 123, 124). To comprehensively assess such temporal patterns in glucose control, continuous glucose monitoring systems (CGMS) are needed, particularly in women with T1DM, as fluctuations are often missed by SMBG (262, 263). Indeed, initial studies using CGMS have shown maternal glucose excursion profiles are related to macrosomia (90, 264–266). For example, a multi-center study involving women with T1DM and T2DM using CGMS, identified that glucose excursions at specific time periods throughout the day were associated with macrosomia (birthweight >90th percentile) in each trimester (264). For the second and third trimesters, the macrosomia-related glucose levels were higher and showed greater variability.

Meanwhile, studies in the setting of GDM have mostly used measures of maternal glucose obtained from the oral glucose tolerance test (OGTT). These measures, particularly the fasting values, have often been associated with macrosomia (95–99). Although, a Canadian study found glucose levels (fasting or post-glucose load) only independently accounted for 3–5% of the variance in birthweight (98).

Importantly, maternal glycaemia has been shown to be related to macrosomia risk amongst healthy women in the absence of overt diabetes (102, 103). The landmark study in the area is the Hyperglycaemia and Adverse Pregnancy Outcomes (HAPO) study. This multinational investigation assessed outcomes associated with glucose parameters from the OGTT at 24–32 weeks' gestation in healthy pregnant women (102). The blinded data from ~23,000 participants demonstrated linear associations between increasing maternal glucose levels below diagnostic thresholds for diabetes with both birthweight above the 90th percentile and umbilical cord blood C-peptide above the 90th percentile (indicative of fetal hyperinsulinaemia). The findings support the Pedersen hypothesis and indicated even mild maternal hyperglycaemia without diabetes increases macrosomia risk, which has had subsequent implications for GDM diagnostic thresholds.

However, other studies with participants of varying diabetes status have not found blood glucose to be associated with birthweight (25, 109–111). Variations in glucose testing protocols and treatment regimens may provide some explanation for this. Thus, uncertainties remain regarding the utility of blood glucose for birthweight/macrosomia prediction (8).

#### Glycosylated hemoglobin (HbA1c)

HbA1c is produced by non-enzymatic glycosylation of hemoglobin. It is a long-term marker of glycaemic control, reflecting the average glucose concentration over the previous 2–3 months (267, 268).

In women with pre-existing diabetes, macrosomia and birthweight have been significantly associated with HbA1c measured at different time points. In multiple studies, third trimester values have demonstrated positive associations with macrosomia (90, 92, 109, 123, 126, 127) and birthweight (91, 128). A notable study is a prospective nation-wide investigation of 289 women with T1DM in The Netherlands (123). Amongst this cohort with acceptable glycaemic control, the third trimester HbA1c measurement was the strongest predictor of macrosomia (birthweight >90th percentile), accounting for 4.7% of the variance of macrosomia. Furthermore, HbA1c measured in trimester 1 (119, 121), or trimester 2 and 3 (116, 124, 125, 129) have also been associated with birthweight or macrosomia. Across all of these studies, HbA1c has been reported to explain ~5–23% of variance in birthweight (119, 123).

Contrastingly, other research groups have not found a significant association between HbA1c and birthweight (19, 87, 88, 269). Contributing factors to these inconsistencies may include the sample collection time-points and the use of averages of HbA1c across varying periods. This could be influential as HbA1c normally declines during pregnancy and is a retrospective weighted average marker (267). It may also relate to the study protocol reducing glycaemic variability (87, 88) or to differences in analytical assays (270).

In addition, a prominent issue is the persistence of high macrosomia rates in women with pre-existing diabetes even when HbA1c values indicate “good” glycaemic control (87, 92, 123, 133). This “macrosomia despite normoglycaemia” may be linked to HbA1c-determined normoglycaemia not revealing glycaemic variability. As previously mentioned, postprandial hyperglycaemia and glycaemic fluctuations are considered important in accelerating growth (8, 271). This is consistent with some studies indicating tighter blood glucose control and thus reduced glycaemic excursions, can reduce macrosomia incidence (260, 272, 273).

There is weaker evidence for a significant association between HbA1c and birthweight in women with GDM (21, 95, 111, 134, 135). This may related to the reduced aberrations in HbA1c in GDM compared with T1DM and T2DM (274). Also, many studies have measured HbA1c at the same time as GDM diagnostic testing (around 28 weeks' gestation) for convenience. However, when HbA1c was measured at delivery in women with GDM, HbA1c >6.8% was associated with a fivefold increased risk of macrosomia (birthweight ≥4,000 g) compared with HbA1c<6.0% (81). Later HbA1c testing may therefore be more useful for predicting macrosomia.

Meanwhile, for women without diabetes, a correlation between HbA1c at various times with birthweight has been identified by some (103, 130, 131, 139), but not other researchers (137, 138). In the HAPO cohort, glucose measures had a significantly stronger association with birthweight than HbA1c (132). Furthermore, another study assessed ultrasound and HbA1c prediction of macrosomia (birthweight ≥4,000 g) within 1 week prior to delivery (138). It found HbA1c measurements were not useful and thus could not improve ultrasound prediction accuracy. However, HbA1c levels were low in this cohort without diabetes. Thus overall, HbA1c may be more useful in women with pre-existing diabetes and later in pregnancy.

#### 1,5-anhydroglucitol (1,5-AG)

1,5-AG is the 1-deoxy form of glucose and is a short-term glycaemic marker (275). During normoglycaemia, serum 1,5-AG is in a steady-state, with >99% renal reabsorption (275). However in hyperglycaemic conditions, glucose competitively inhibits renal reabsorption of 1,5-AG, thereby increasing 1,5-AG excretion and reducing the serum 1,5-AG concentration (268). It reflects the glycaemic control over the preceding 24 h to 2 weeks, and importantly, it can capture glycaemic fluctuations (268, 275).

With these benefits in detecting glycaemic excursions, Nowak et al. compared 1,5-AG and HbA1c in pregnant women with T1DM and found 1,5-AG was the stronger predictor of macrosomia (birthweight >90th percentile) (20). The receiver operator characteristic (ROC) area under the curve (AUC) for third trimester 1,5-AG macrosomia prediction was 0.81. This improved to 0.84 with the addition of HbA1c, and could achieve sensitivity and specificity of 86 and 71%, respectively. As 80% of the cases of macrosomia occurred in women that met HbA1c targets, it suggested that glucose excursions that were not reflected in the HbA1c level but were captured by the 1,5-AG values may have contributed to fetal overgrowth. This assertion was supported by CGMS records which showed 1,5-AG was strongly correlated with CGMS indices including a measure of glucose variability, but HbA1c was not.

Building on this, a study involving women with T1DM, T2DM, and GDM found that there was a significant inverse association between 1,5-AG and birthweight *z*-score across the groups (19). Of note, HbA1c was not associated with birthweight, possibly due to the participants having overall low HbA1c measurements. The authors contend however, that as 1,5-AG was significantly associated with birthweight even amongst a population with good glycaemic control according to HbA1c, it could be used to identify the subset of pregnancies that are at risk of macrosomia, despite HbA1c within target ranges.

Wright et al. similarly found an inverse linear association between mean 1,5-AG and birthweight *z*-scores in a cohort of T1DM, T2DM, and GDM pregnancies (18). The association for mean HbA1c was not significant. The lack of blood glucose data (SMBG/CGMS) is a limitation, as conclusions regarding glycaemic control and fluctuations require consideration of these immediate measures of glycaemia.

In contrast, a study comparing women with GDM and pregnant women without diabetes did not find 1,5-AG to be a significant predictor of birthweight (21). Although, serum 1,5-AG concentration was significantly lower in the women with GDM compared to controls and there was a trend for an inverse association between 1,5-AG and birthweight in the GDM group.

There have been concerns that the reduction in renal glucose threshold during normal pregnancy may affect renal excretion of 1,5-AG and thus serum levels, thereby possibly limiting the utility of 1,5-AG in reflecting glycaemic changes while pregnant (275, 276). However, the few available evaluations of 1,5-AG as a marker of glycaemic control in pregnancies complicated by diabetes have shown that it performs well (20, 277).

#### Lipids

Lipid metabolism is altered during normal pregnancy. Increased fat storage occurs initially in the “anabolic phase” of pregnancy (278). The switch to the “catabolic phase” in the third trimester involves prominent lipolysis promoted by insulin resistance (279, 280). This is paralleled with an increase in the major lipid fractions, predominantly triglycerides (220, 278); which is seen to a greater extent in women with diabetes (278, 281). In accordance with the modified Pedersen hypothesis, maternal lipids may be an important fuel in fetal overgrowth (220).

Of all lipids, triglycerides have been most consistently related to birthweight in pregnancies with diabetes. A study comparing women with T1DM, T2DM, and controls found both third trimester triglycerides and high-density lipoprotein cholesterol (HDL-C) were significantly associated with macrosomia (birthweight >90th percentile), independent of maternal glycaemic control (143). In GDM pregnancies, maternal triglycerides have also been identified as a predictor of macrosomia independent of maternal BMI and glycaemic control (144, 209). Moreover, the ratio of triglycerides to HDL-C has also been examined in women with well-controlled GDM and women without diabetes at 24–28 weeks' gestation (282). The ROC AUC for macrosomia (birthweight >90th percentile) prediction was 0.668, which increased to 0.806 when combined with HbA1c and pre-pregnancy BMI. However, the overall prevalence of macrosomia was low in this population. Together these results indicate lipid alterations may play a distinct role in macrosomia development amongst women with diabetes. Indeed, maternal lipids have been proposed as a potential key factor in “macrosomia despite normoglycaemia” (10, 283).

In women without diabetes, second or third trimester maternal triglycerides have been found to be positively associated with birthweight (25, 211), as well as an independent predictor of macrosomia (94, 146, 207, 212, 213). In a Japanese cohort, maternal fasting hypertrigylceridaemia significantly independently predicted macrosomia (birthweight >90th percentile), with an odds ratio of 11.6 (214). Notably, triglycerides were more strongly associated with fetal growth than maternal glycaemia; although, the small sample size (146 people) may have limited analysis. It is supported however by a similar finding in a cohort that included women with GDM (208). In this study, the triglyceride concentrations after an OGTT were independently associated with birthweight and also predicted glucose intolerance.

Furthermore, macrosomia risk, and birthweight has been inversely associated with second and third trimester maternal HDL-C concentrations in women with pre-existing diabetes (143) and GDM or healthy pregnancies (55, 100, 111, 146, 147). In Zhou et al.'s study which included GDM pregnancies, low HDL-C (<2.2 mmol/L) at 20 weeks' gestation predicted macrosomia (birthweight >4,000 g) with 65% sensitivity and 48% specificity (55).

In contrast, other lipid parameters have less supportive evidence. In women without diabetes, very-low density lipoprotein cholesterol (VLDL-C) has been negatively associated with birthweight (57). While low-density lipoprotein cholesterol (LDL-C) has mostly demonstrated non-significant results (21, 30, 55, 143).

Overall, these studies suggest differential importance of maternal lipid fractions for fetal growth. Again, some studies have not found an association between lipids and birthweight or macrosomia in diabetic or healthy pregnancies (21, 30). Measurement timing may be relevant to this due to the changes in lipid profile and hence possibly their role across pregnancy.

#### Adiponectin

Adiponectin is an adipokine—a bioactive peptide derived from adipose tissue (284). It has important roles in regulating insulin sensitivity and metabolism, and is inversely related to adipose mass and insulin resistance (284, 285). Given this, adiponectin is a possible mediator in the link between maternal adiposity, insulin resistance, and excessive fetal growth (284, 286). Maternal adiponectin may influence fetal growth via altering placental substrate transport as it does not traverse the placenta (286).

There is a notable lack of investigation of maternal adiponectin in women with pre-existing diabetes. Fetal adiponectin however, has received some attention. A comparison of neonates from mothers with T1DM with healthy controls found umbilical cord blood adiponectin collected at birth was not associated with birthweight (23). Similarly, cord blood adiponectin was not associated with birthweight in a study examining offspring of women with T2DM, GDM, and controls (46).

Amongst GDM pregnancies there have been variable results. Tsai and associates compared maternal adiponectin levels collected between 24 and 31 weeks' gestation in women with GDM and controls (26). Adiponectin was significantly lower in the GDM women, and a negative association between maternal adiponectin and birthweight was evident but only significant for the GDM group. Pre-pregnancy BMI ≥27 was also associated with lower adiponectin levels. Cseh et al. corroborated that maternal plasma adiponectin is significantly lower in women with GDM compared to non-diabetic pregnant women and age-matched non-diabetic non-pregnant women (27). Contrastingly though, a significant positive linear correlation was demonstrated between maternal plasma adiponectin and birthweight corrected for gestational age in both the GDM and non-diabetic groups. Meanwhile, others have not found an association between adiponectin and birthweight (36, 37). These conflicting findings may be related to differences between the populations, including BMI, ethnicity, and GDM management, as well as timing of the samples. The adiponectin fractions assessed may also be relevant, as one research group found that only the middle molecular weight isoforms were significantly negatively associated with birthweight in GDM (28).

Verhaeghe et al. also evaluated the value of metabolic biomarkers for birthweight prediction in GDM and control women (29). Maternal adiponectin combined with four other metabolic markers together added 2% to the ~10% explained birthweight variance from maternal body size parameters alone. However, only a single measurement was taken at 24–29 weeks' gestation and given insulin resistance is maximal in the third trimester, greater utility may be provided with later testing.

Findings from healthy pregnancies also provide insight. In two case-control studies involving women without diabetes, macrosomia groups had significantly lower maternal adiponectin concentrations compared with the controls (31, 32). In one of these, maternal adiponectin measured between 11 and 13 weeks' gestation improved macrosomia (birthweight >95th percentile) detection to 38.2% when added to maternal characteristics and obstetric history (compared with 34.6% without adiponectin) (31). Moreover, an independent inverse relationship was identified between maternal adiponectin and birthweight in a subset of the HAPO cohort (34). Although, other studies have not found a significant association with maternal adiponectin (38, 39).

Thus, despite inconsistencies there is indication adiponectin may be related to birthweight. Pre-existing diabetes is an area that particularly requires further investigation.

#### Insulin-like growth factor-1 (IGF-1)

IGF-1 is a peptide hormone principally produced in the liver (287). Normal pregnancy involves changes in the maternal IGF axis, including variations to IGF-binding proteins (IGFBPs) (287). Consequently, this results in increased free IGF-1, the biologically active form. With mitogenic and metabolic actions, maternal and fetal IGF-1 are believed to be important mediators in fetal growth (285, 287). For maternal IGF-1, this may be via its role in regulating transplacental nutrient transport (288, 289).

However, the literature on maternal IGF-1 in pre-existing diabetes is conflicting. In a prospective study involving serial maternal IGF-1 measurements in women with pre-existing diabetes, IGF-1 was significantly positively associated with macrosomia (156). Yet in a similar study involving only women with T1DM, there were no differences in maternal IGF-1 across pregnancy in diabetic women delivering a macrosomic neonate compared with appropriate birthweight neonate (126). A notable difference between the studies were the methods of macrosomia assessment. The first study created *post-hoc* groupings according to birthweight ratio whereas the second used the definition of birthweight >90th percentile. Likewise, another research group found macrosomia according to this latter definition was not significantly associated with maternal IGF-1 (142). Although, they did show maternal IGF-1 measured at 36 weeks' gestation was significantly associated with birthweight *z*-score (142). Thus, the assessment measure of birthweight/macrosomia may be relevant in identifying an association with a biomarker.

GDM pregnancies have also been investigated. In a study of GDM and control women, elevated IGF-1 in maternal blood in mid- and late- gestation and fetal cord blood at birth predicted macrosomia (birthweight >90th percentile) (159). This is consistent with a case-control study in which serum IGF-1 levels were significantly higher in the GDM women and their macrosomic neonates (birthweight >2 standard deviations) compared to matched controls with appropriate birthweight neonates (290). Other studies have also found IGF-1 related to birthweight (174, 291).

The relationship of IGF-1 to fetal growth across the different types of diabetes requires clarification. An investigation comparing women with T1DM, T2DM, GDM, and controls and found the third trimester median maternal IGF-1 values were not significantly different between the groups (157). Considering all the women with diabetes together, third trimester IGF-1 was positively associated with birthweight percentile, explaining 24% of the variation in birthweight. However, other reports suggest the changes across pregnancy in IGF-1 for pre-existing diabetes and GDM are different, with lower levels in pre-existing diabetes (163, 164, 177) and higher levels in GDM (258) compared with controls.

For pregnancies without diabetes, maternal IGF-1 (161, 162, 167) and fetal IGF-1 (38, 161, 166, 175, 176) have been associated with birthweight and macrosomia. These studies have been conducted in populations of various ethnicities. Non-significant associations have also been reported (165, 168, 178, 292). Such discrepancies may relate to factors such as the method of IGF-1 analysis, timing of measurements, or the sample size (287).

Altogether the evidence is conflicting. Adequate assessment has not been made of the predictive utility of IGF-1 for birthweight and macrosomia in women with diabetes.

### Evaluation of macrosomia biomarkers

Biomarkers face many challenges in becoming adopted into routine clinical practice. One of the most important requirements is biomarker validation (16), which is particularly an issue in the field of macrosomia biomarkers. In this area, substantial variability exists amongst published results, with the heterogeneity of the studies a prominent contributor. Indeed, there is considerable variation in study designs, populations, measurement timing, outcome variables (including macrosomia definitions), and analytical methods. This limits the conclusions that can be made at this time and highlights the need for rigorous validation protocols to comprehensively evaluate macrosomia biomarker predictive performance. Future work in this field must also assess the cost-effectiveness of biomarker use. Biomarker adoption may become more feasible with improved accessibility of commercial biomarker kits and multiparametric biomarker testing for multiple pregnancy disorders together (e.g., with preeclampsia biomarkers as they also become validated).

Furthermore, as biomarkers may be useful as part of a combination approach, whereby biomarkers are incorporated into a prediction algorithm with other elements such as macrosomia risk factors, physical examination and ultrasound measurements, further investigation is also needed that compares biomarkers to and in combination with the other methods of macrosomia prediction. Of the limited such assessments available, biomarkers have improved predictive performance when combined with risk factors (29, 31, 282) but not ultrasound (138). A further step is to determine if improved macrosomia predictive accuracy can improve clinical outcomes.

## Conclusion

Accurately predicting fetal macrosomia remains a desirable but challenging goal. While biomarkers hold promise for assisting in this plight, the current state of knowledge for macrosomia biomarkers is limited. The selected biomarkers in this review each have a theoretical link with macrosomia with some supportive evidence for an association with birthweight/macrosomia. However, due to the limitations of the literature, the true value of biomarkers is not yet clear. Further research is needed to address this, particularly in pregnancies affected by diabetes. A focus on this area is warranted as there is great potential and much to be gained by further exploration. Indeed, broader implications of this research includes providing greater insight into the pathophysiological processes of excessive growth; which is of special interest due to the links with later development of chronic disease (Barker hypothesis). Ultimately, improving outcomes for pregnant women and their babies is the driving force for this research.

## Author contributions

SN was the primary author. EE, JS, and AS edited the manuscript. CH reviewed the manuscript.

### Conflict of interest statement

The authors declare that the research was conducted in the absence of any commercial or financial relationships that could be construed as a potential conflict of interest.
