# Supplementary material for: Biomarkers for Macrosomia Prediction in Pregnancies Affected by Diabetes
Source: Front Endocrinol (Lausanne). 2018 Jul 31;9:407. doi: 10.3389/fendo.2018.00407 (PMC6079223; doi:10.3389/fendo.2018.00407)
Supplement: Supplementary file 1 [file Table_1.PDF]

Supplementary table 1: Macrosomia definitions		
**Biomarker**	**Paper**	**Macrosomia definition**
Blood glucose	Jovanovic-Peterson L, Peterson CM, Reed GF, Metzger BE, Mills JL, Knopp RH, et al. Maternal postprandial glucose levels and infant birth weight: the Diabetes in Early Pregnancy Study. American Journal of Obstetrics & Gynecology. 1991;164(1):103-11.	Birthweight ≥ 90th percentile for gestational age, sex, and race.
	Combs CA, Gunderson E, Kitzmiller JL, Gavin LA, Main EK. Relationship of fetal macrosomia to maternal postprandial glucose control during pregnancy. Diabetes Care. 1992;15(10):1251-7.	Birthweight >90th percentile for gestational age based on California norms.
	Persson B, Hanson U. Fetal size at birth in relation to quality of blood glucose control in pregnancies complicated by pregestational diabetes mellitus. British Journal Of Obstetrics And Gynaecology. 1996;103(5):427-33.	Birthweight > 2 standard deviations from the national normal mean after correction for gestational age and sex.
	Law GR, Ellison GTH, Secher AL, Damm P, Mathiesen ER, Temple R, et al. Analysis of continuous glucose monitoring in pregnant women with diabetes: distinct temporal patterns of glucose associated with large-for-gestational-age infants. Diabetes Care. 2015;38(7):1319-25.	Birthweight ≥ 90th percentile for sex and gestational-adjusted birthweight according to British and Scandinavian growth references.
	Kerssen A, de Valk HW, Visser GHA. Increased second trimester maternal glucose levels are related to extremely large-for-gestational-age infants in women with type 1 diabetes. Diabetes Care. 2007;30(5):1069-74.	Birthweight ≥ 90th percentile after correction for gestational age, sex, and parity, according to Dutch growth charts.
	Murphy HR, Rayman G, Lewis K, Kelly S, Johal B, Duffield K, et al. Effectiveness of continuous glucose monitoring in pregnant women with diabetes: randomised clinical trial. British Medical Journal. 2008;337(7675):907-10.	Birthweight ≥ 90th percentile for gestational age and sex.
	Shen S, Lu J, Zhang L, He J, Li W, Chen N, et al. Single fasting plasma glucose versus 75-g oral glucose-tolerance test in prediction of adverse perinatal outcomes: a cohort study. EBioMedicine. 2017;16:284-91.	Birthweight > 90th percentile for gestational age by sex according to a local population-based birthweight reference.
	Disse E, Graeppi-Dulac J, Joncour-Mills G, Dupuis O, Thivolet C. Heterogeneity of pregnancy outcomes and risk of LGA neonates in Caucasian females according to IADPSG criteria for gestational diabetes mellitus. Diabetes & Metabolism. 2013;39(2):132-38.	Birthweight >90th percentile for gestational age.
	Ouzilleau C, Roy MA, Leblanc L, Carpentier A, Maheux P. An observational study comparing 2- hour 75-g oral glucose tolerance with fasting plasma glucose in pregnant women: both poorly predictive of birth weight. Canadian Medical Association Journal. 2003;168(4):403-9.	Birthweight ≥ 90th percentile for gestational age and sex according to Canadian standards.
	Nasrat H, Fageeh W, Abalkhail B, Yamani T, Ardawi MS. Determinants of pregnancy outcome in patients with gestational diabetes. International Journal Of Gynaecology And Obstetrics. 1996;53(2):117- 23.	Birthweight >4000 grams.
	Lapolla A, Dalfrà MG, Bonomo M, Castiglioni MT, Di Cianni G, Masin M, et al. Can plasma glucose and HbA1c predict fetal growth in mothers with different glucose tolerance levels? Diabetes Research and Clinical Practice. 2007;77:465-70.	Macrosomia: birthweight >4000grams.<br>Large for gestational age: birthweight >90th percentile for their population.
	The HAPO Study Cooperative Research Group. Hyperglycemia and adverse pregnancy outcomes. New England Journal of Medicine. 2008;358(19):1991-2002.	Birthweight >90th percentile for gestational age.
	Schrader HM, Jovanovic-Peterson L, Bevier WC, Peterson CM. Fasting plasma glucose and glycosylated plasma protein at 24 to 28 weeks of gestation predict macrosomia in the general obstetric population. American Journal Of Perinatology. 1995;12(4):247-51.	Birthweight >4000 grams.

Glycosylated haemoglobin	Evers IM, de Valk HW, Mol BWJ, ter Braak EWMT, Visser GHA. Macrosomia despite good glycaemic control in Type I diabetic pregnancy; results of a nationwide study in The Netherlands. Diabetologia. 2002;45(11):1484-9.	Birthweight >90th percentile corrected for gestational age, sex, parity, and race, according to Dutch growth charts.
	Herranz L, Pallardo LF, Hillman N, Martin-Vaquero P, Villarroel A, Fernandez A. Maternal third trimester hyperglycaemic excursions predict large-for-gestational-age infants in type 1 diabetic pregnancy. Diabetes Research and Clinical Practice. 2007;75:42-6.	Birthweight >90th percentile using a specific growth chart (Battaglia and Lubchenco, Journal of Pediatrics 1967).
	Kerssen A, de Valk HW, Visser GHA. Increased second trimester maternal glucose levels are related to extremely large-for-gestational-age infants in women with type 1 diabetes. Diabetes Care. 2007;30(5):1069-74.	Birthweight ≥ 90th percentile corrected for gestational age, sex, and parity, according to Dutch growth charts.
	Berk MA, Mimouni F, Miodovnik M, Hertzberg V, Valuck J. Macrosomia in infants of insulin-dependent diabetic mothers. Pediatrics. 1989;83(6):1029-34.	Birthweight >90th percentile of the intrauterine growth curves of Lubchenco (Lubchenco et al., Pediatrics 1979).
	Ringholm L, Juul A, Pedersen-Bjergaard U, Thorsteinsson B, Damm P, Mathiesen ER. Lower levels of placental growth hormone in early pregnancy in women with type 1 diabetes and large for gestational age infants. Growth Hormone & IGF Research. 2015;25(6):312-5.	Birthweight >90th percentile adjusted for gestational age and sex.
	Damm P, Mersebach H, Råstam J, Kaaja R, Hod M, McCance DR, et al. Poor pregnancy outcome in women with type 1 diabetes is predicted by elevated HbA1c and spikes of high glucose values in the third trimester. The Journal Of Maternal-Fetal & Neonatal Medicine. 2014;27(2):149-54.	Macrosomia: birthweight >4000 grams.<br>Large for gestational age: birthweight >90th percentile according to local growth charts.
	Rey E, Attié C, Bonin A. The effects of first-trimester diabetes control on the incidence of macrosomia. American Journal of Obstetrics and Gynecology. 1999;181:202-6.	Birthweight >90th percentile for Canadian norms.
	Johnstone FD, Mao JH, Steel JM, Prescott RJ, Hume R. Factors affecting fetal weight distribution in women with type I diabetes. BJOG: An International Journal Of Obstetrics And Gynaecology. 2000;107(8):1001-6.	Birthweight >95th percentile
	Maresh MJA, Holmes VA, Patterson CC, Young IS, Pearson DWM, Walker JD, et al. Glycemic targets in the second and third trimester of pregnancy for women with type 1 diabetes. Diabetes Care. 2015;38(1):34-42.	Birthweight >90th percentile according to customised birthweight charts.
	Lepercq J, Taupin P, Dubois-Laforgue D, Duranteau L, Lahlou N, Boitard C, et al. Heterogeneity of fetal growth in type 1 diabetic pregnancy. Diabetes & Metabolism. 2001;27(3):339-44.	Ponderal index >90th percentile
	Mello G, Parretti E, Mecacci F, La Torre P, Cioni R, Cianciulli D, et al. What degree of maternal metabolic control in women with type 1 diabetes is associated with normal body size and proportions in full-term infants? Diabetes Care. 2000;23(10):1494-8.	Birthweight ≥ 90th percentile based on the local population growth standards.
	Lin CC, River J, River P, Blix PM, Moawad AH. Good diabetic control early in pregnancy and favorable fetal outcome. Obstetrics & Gynecology. 1986;67:51-6.	Birthweight ≥ 90th percentile for gestational age.
	Cahill AG, Tuuli MG, Colvin R, Cade WT, Macones GA. Markers of glycemic control and neonatal morbidity in high-risk insulin-resistant pregnancies. American Journal Of Perinatology. 2016;33(2):151-6.	Birthweight ≥4000 grams.
	Weissmann-Brenner A, O'Reilly-Green C, Ferber A, Divon MY. Does the availability of maternal HbA1c results improve the accuracy of sonographic diagnosis of macrosomia? Ultrasound In Obstetrics & Gynecology. 2004;23(5):466-71	Birthweight ≥4000 grams.
1,5-Anhydroglucitol	Nowak N, Skupien J, Cyganek K, Matejko B, Malecki MT. 1,5-Anhydroglucitol as a marker of maternal glycaemic control and	Birthweight >90th percentile according to sex for the Polish population.

	predictor of neonatal birthweight in pregnancies complicated by type 1 diabetes mellitus. Diabetologia. 2013;56(4):709-13.	
	Swierzewska P, Kosiński M, Wójcik M, Dworacka M, Cypryk K. Family, anthropometric and biochemical factors affecting birth weight of infants born to GDM women. Ginekologia Polska. 2015;86(7):499-503.	Macrosomia: birthweight >4000 grams.<br>Large for gestational age: birthweight >90th percentile.
Lipids	Gobl CS, Handisurya A, Klein K, Bozkurt L, Luger A, Bancher-Todesca D, et al. Changes in serum lipid levels during pregnancy in type 1 and type 2 diabetic subjects. Diabetes Care. 2010;33(9):2071-3.	Birthweight >90th percentile adjusted for sex and age of the Austrian population.
	Simeonova-Krstevska S, Krstevska B, Velkoska-Nakova V, Hadji Lega M, Samardjiski I, Serafimoski V, et al. Effect of lipid parameters on foetal growth in gestational diabetes mellitus pregnancies. Prilozi. 2014;35(2):131-6.	Birthweight >90th percentile.
	Schaefer-Graf UM, Graf K, Kulbacka I, Kjos SL, Dudenhausen J, Vetter K, et al. Maternal lipids as strong determinants of fetal environment and growth in pregnancies with gestational diabetes mellitus. Diabetes Care. 2008;31(9):1858-63.	Birthweight >90th percentile according to gestational age and sex based on a national German database.
	Wang D, Xu S, Chen H, Zhong L, Wang Z. The associations between triglyceride to high-density lipoprotein cholesterol ratios and the risks of gestational diabetes mellitus and large-for-gestational-age infant. Clinical Endocrinology. 2015;83(4):490-7.	Birthweight >90th percentile for gestational age and sex.
	Hou RL, Zhou HH, Chen XY, Wang XM, Shao J, Zhao ZY. Effect of maternal lipid profile, C-peptide, insulin, and HBA1c levels during late pregnancy on large-for-gestational age newborns. World Journal of Pediatrics. 2014;10(2):175-81	Birthweight >90th percentile for gestational age.
	Mossayebi E, Arab Z, Rahmaniyan M, Almassinokiani F, Kabir A. Prediction of neonates' macrosomia with maternal lipid profile of healthy mothers. Pediatrics & Neonatology. 2013;55(1):28-34.	Macrosomia: birthweight ≥4000 grams.<br>Large for gestational age: birthweight >75th percentile for gestational age.
	Di Cianni G, Miccoli R, Volpe L, Lencioni C, Ghio A, Giovannitti MG, et al. Maternal triglyceride levels and newborn weight in pregnant women with normal glucose tolerance. Diabetic Medicine. 2004;22(1):21-5.	Macrosomia: birthweight ≥4000 grams.<br>Large for gestational age: birthweight >90th percentile for gestational age.
	Jin W-Y, Lin S-L, Hou R-L, Chen X-Y, Han T, Jin Y, et al. Associations between maternal lipid profile and pregnancy complications and perinatal outcomes: a population-based study from China. BMC Pregnancy & Childbirth. 2016;16:1-9.	Macrosomia: birthweight >4000 grams.<br>Large for gestational age: birthweight >90th percentile for gestational age.
	Ahmad SM, Hazlina NH, Che Anuar CY, Faridah AR, Shukri Y. A study on factors affecting newborn weight and large for gestational age (LGA) newborns in non-diabetic mothers: the role of maternal serum triglycerides. International Medical Journal. 2006;13(1):53-8.	Birthweight >90th percentile for gestational age and sex, for Malaysian population.
	Kitajima M, Oka S, Yasuhi I, Fukuda M, Rii Y, Ishimaru T. Maternal serum triglyceride at 24-32 weeks' gestation and newborn weight in nondiabetic women with positive diabetic screens. Obstetrics & Gynecology. 2001;97:776-80	Birthweight >90th percentile for sex-specific Japanese weight curves.

	Krstevska B, Velkoska NV, Adamova G, Simeonova S, Dimitrovski C, Livrinova V, et al. Association between foetal growth and different maternal metabolic characteristics in women with gestational diabetes mellitus. Contributions of Macedonian Academy of Sciences & Arts. 2009;30(2):103.	Birthweight >90th percentile for gestational age and sex for the local population.
	Clausen T, Burski TK, Øyen N, Godang K, Bollerslev J, Henriksen T. Maternal anthropometric and metabolic factors in the first half of pregnancy and risk of neonatal macrosomia in term pregnancies. A prospective study. European Journal Of Endocrinology. 2005;153(6):887-94.	Birthweight >4500grams or z-score >95th percentile according to gestational age and sex, based on an Norwegian database.
	Zhou J, Zhao X, Wang Z, Hu Y. Combination of lipids and uric acid in mid-second trimester can be used to predict adverse pregnancy outcomes. Journal of Maternal-Fetal & Neonatal Medicine. 2012;25(12):2633-8.	Birthweight >4000 grams.
	Retnakaran R, Ye C, Hanley AJG, Connelly PW, Sermer M, Zinman B, et al. Effect of maternal weight, adipokines, glucose intolerance and lipids on infant birth weight among women without gestational diabetes mellitus. Canadian Medical Association Journal 2012;184(12):1353-60.	Macrosomia: birthweight ≥4000 grams.<br>Large for gestational age: birthweight >90th percentile for gestational age, ethnicity and sex for Canadian growth curves.
Adiponectin	Nanda S, Akolekar R, Sarquis R, Mosconi AP, Nicolaides KH. Maternal serum adiponectin at 11 to 13 weeks of gestation in the prediction of macrosomia. Prenatal Diagnosis. 2011;31(5):479-83.	Birthweight >95th percentile for gestational age.
	Wang J, Shang L, Dong X, Wang X, Wu N, Wang S, et al. Relationship of adiponectin and resistin levels in umbilical serum, maternal serum and placenta with neonatal birth weight. Australian & New Zealand Journal of Obstetrics & Gynaecology. 2010;50(5):432-8	Birthweight >4000 grams.
Insulin-like growth factor-1	Lauszus FF, Klebe JG, Flyvbjerg A. Macrosomia associated with maternal serum insulin-like growth factor-I and -II in diabetic pregnancy. Obstetrics & Gynecology. 2001;97:734-41.	Levels of macrosomia:<br>- birthweight ratio <1.18<br>- birthweight ratio 1.18-1.4<br>- birthweight ratio >1.4<br><br>(birthweight ratio = actual birthweight ÷ expected birthweight for the gestational age and sex based in local population)
	Ringholm L, Juul A, Pedersen-Bjergaard U, Thorsteinsson B, Damm P, Mathiesen ER. Lower levels of placental growth hormone in early pregnancy in women with type 1 diabetes and large for gestational age infants. Growth Hormone & IGF Research. 2015;25(6):312-5.	Birthweight >90th percentile for gestational age and sex.
	McIntyre HD, Serek R, Crane DI, Veveris-Lowe T, Parry A, Johnson S, et al. Placental growth hormone (GH), GH-binding protein, and insulin-like growth factor axis in normal, growth-retarded, and diabetic pregnancies: correlations with fetal growth. The Journal Of Clinical Endocrinology And Metabolism. 2000;85(3):1143-50.	Birthweight >90th percentile for gestational age and sex.
	Luo Z-C, Nuyt A-M, Delvin E, Audibert F, Girard I, Shatenstein B, et al. Maternal and fetal IGF-I and IGF-II levels, fetal growth, and	Birthweight >90th percentile for gestational age and sex according to Canadian growth charts.

	gestational diabetes. The Journal of Clinical Endocrinology & Metabolism. 2012;97(5):1720-8.	
	Grissa O, Yessoufou A, Mrisak I, Hichami A, Amoussou-Guenou D, Grissa A, et al. Growth factor concentrations and their placental mRNA expression are modulated in gestational diabetes mellitus: possible interactions with macrosomia. BMC Pregnancy and Childbirth. 2010;10(1):7.	Birthweight > 2 standard deviations from the mean birthweight of the control infants.
	Osorio M, Torres J, Moya F, Pezzullo J, Salafia C, Baxter R, et al. Insulin-like growth factors (IGFs) and IGF binding proteins-1, -2, and -3 in newborn serum: relationships to fetoplacental growth at term. Early Human Development. 1996;46(1-2):15-26.	Birthweight >90th percentile for gestational age.
	Chiesa C, Osborn JF, Haass C, Natale F, Spinelli M, Scapillati E, et al. Ghrelin, leptin, IGF-1, IGFBP-3, and insulin concentrations at birth: is there a relationship with fetal growth and neonatal anthropometry? Clinical chemistry. 2008;54(3):550-8.	Birthweight >90th percentile for gestational age and sex according to Italian growth charts.
